# Synthesis and Characterization of Molecularly Imprinted Polymer Membrane for the Removal of 2,4-Dinitrophenol

**DOI:** 10.3390/ijms14023993

**Published:** 2013-02-18

**Authors:** Nor Azah Yusof, Nor Dyana Zakaria, Nor Amirah Mohd Maamor, Abdul Halim Abdullah, Md. Jelas Haron

**Affiliations:** 1 Department of Chemistry, Faculty of Science, Universiti Putra Malaysia, Serdang 43400, Selangor, Malaysia; E-Mails: nordyana.zakaria@gmail.com (N.D.Z.); amiramaamor@gmail.com (N.A.M.M.); halim@science.upm.edu.my (A.H.A.); mdjelas@science.upm.edu.my (M.J.H.); 2 Institute of Advanced Technology (ITMA), Universiti Putra Malaysia, Serdang 43400, Selangor, Malaysia; 3 Institute for Research in Molecular Medicine (INFORMM), Universiti Sains Malaysia, Pulau Pinang 11800, Malaysia

**Keywords:** molecular imprinted polymer, 2,4-dinitrophenol cellulose acetate, polysulfone

## Abstract

Molecularly imprinted polymers (MIPs) were prepared by bulk polymerization in acetonitrile using 2,4-dinitrophenol, acrylamide, ethylene glycol dimethacrylate, and benzoyl peroxide, as the template, functional monomer, cross-linker, and initiator, respectively. The MIP membrane was prepared by hybridization of MIP particles with cellulose acetate (CA) and polystyrene (PS) after being ground and sieved. The prepared MIP membrane was characterized using Fourier transform infrared spectroscopy and scanning electron microscopy. The parameters studied for the removal of 2,4-dinitrophenol included the effect of pH, sorption kinetics, and the selectivity of the MIP membrane. Maximum sorption of 2,4-nitrophenol by the fabricated CA membrane with MIP (CA-MIP) and the PS membrane with MIP (PS-MIP) was observed at pH 7.0 and pH 5.0, respectively. The sorption of 2,4-dinitrophenol by CA-MIP and PS-MIP followed a pseudo–second-order kinetic model. For a selectivity study, 2,4-dichlorophenol, 3-chlorophenol, and phenol were selected as potential interferences. The sorption capability of CA-MIP and PS-MIP towards 2,4-dinitrophenol was observed to be higher than that of 2,4-dichlorophenol, 3-chlorophenol, or phenol.

## 1. Introduction

Water pollution causes many problems for individual organisms, populations, and communities. The relationship between water quality and human activity is very complicated. Water used in domestic, industrial, and agricultural sectors is usually returned to rivers, lakes, estuaries, or oceans; however, industrial effluent is usually polluted by organic compounds, phenolic compounds and their derivatives being the most common. Nitrophenols are considered priority toxic pollutants by the U.S. Environmental Protection Agency and are therefore of particular interest [1].

Various methods are available to remove nitrophenols from water, including adsorption [2–6], microbial degradation [7], chemical oxidation [8], membrane separation [9], catalytic oxidation [10], and electrochemical treatment [11,12]. Some of these methods, such as adsorption on activated carbon, have high costs, as well as the potential to cause secondary pollution, and low-cost, highly selective removal methods continue to be sought [1].

Molecularly imprinted polymers (MIPs) are being utilized in an increasing number of applications, as “tailor-made” separation materials, antibody–receptor binding site mimics in recognition and assay systems, enzyme mimics in catalytic applications, recognition elements in sensors, and in facilitated chemical synthesis [13–15]. To date, their most extensively investigated application has been as separation materials for the analysis of various compounds, including drugs [16,17], pesticides [18,19], and amino acids [20]. A highly specific detection technology, MIPs have been used for the separation of isomers and enantiomers [21], solid extraction [22], in biochemical sensors [23] and chemosensors [24], in simulating enzyme-catalysed pharmaceutical analysis [25], in sorbents, and in membrane separation technologies [26]. They have been prepared in various configurations—including polymer beads, monoliths, and membranes—and have numerous advantages, such as physical robustness, high strength, resistance to elevated temperatures and pressures, and inertness towards organic solvents, acids, and bases [27]. Further, MIPs are stable, easy to prepare, and inexpensive.

Investigations of MIP membranes have been increasing in the last few years. Several articles have been published on the preparation of MIP membranes, showing specific permeability and separation for template/ligands such as cholesterol [28], nucleotides, and various drugs [29]. The transport properties and applications of MIP membranes in sensor technology have also been investigated.

The present study used a MIP membrane as a sorbent for the removal of 2,4-dinitrophenol (2,4-DNP) from aqueous solution. The aim of this study is to investigate the adsorption of 2,4-DNP by the MIP membrane, specifically using cellulose acetate (CA) and polysulfone (PSf). To the best of our knowledge, this work is the first to report on the development of an MIP membrane for the removal of 2,4-nitrophenol. The parameters studied include pH, sorption kinetics, and sorption isotherms. A selectivity study is also conducted. The prepared MIP membrane can also be used for the separation, enrichment, and trace analysis of targeted phenolic compounds in aqueous samples.

## 2. Results and Discussion

### 2.1. Characterization of CA-MIP and PS-MIP Membranes

#### 2.1.1. Fourier Transform Infrared Spectroscopy (FTIR) Spectra of CA-MIP and PS-MIP

**Figure 1:**
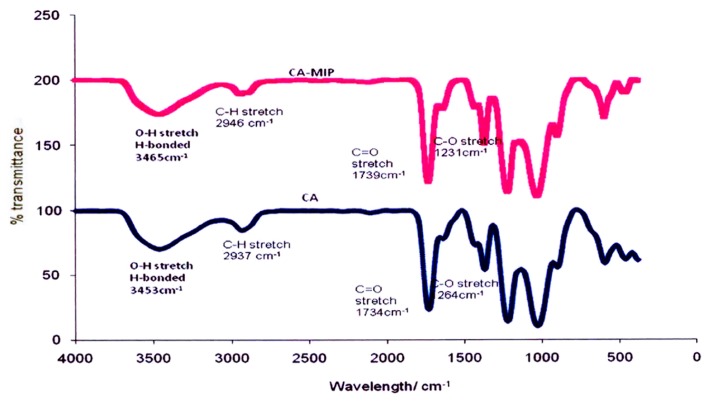
FTIR spectra for CA and CA-MIP.

**Figure 2:**
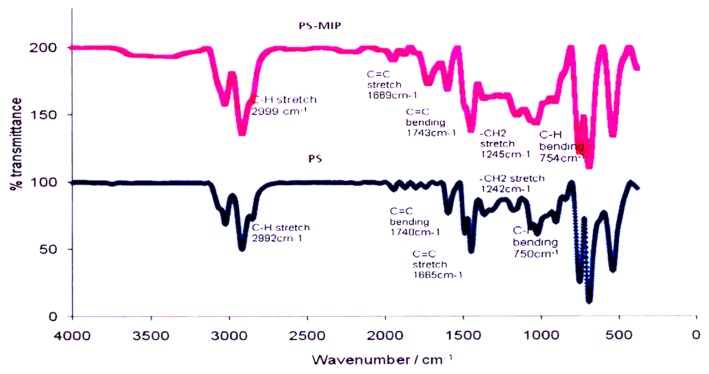
FTIR spectra for PS and PS-MIP.

Figure 1 shows the Fourier transform infrared spectroscopy (FTIR) spectra for a CA membrane and CA-MIP, and Figure 2 shows the FTIR spectra of a PS membrane and PS-MIP. After the CA membrane is imprinted, the CH stretching of the CA membrane band in CA-MIP has clearly shifted towards a higher wavenumber, from 2937 cm^−1^ to 2946 cm^−1^. Furthermore, the shift of the C=O stretching band of the CA membrane at 1734 cm^−1^ to the higher wavenumber, 1739 cm^−1^, is also strong evidence for hybridization of MIP into the CA membrane. In the PS-MIP, it was found that the C=C and −CH^2^ stretching bands were shifted from 1665 cm^−1^ to 1669 cm^−1^, and from 1242 cm^−1^ to 1245 cm^−1^, respectively. The band shifts are evidence of hybridization of the imprinted polymer particles in the PS membrane.

Takeda *et al.* [30] made the same observation, using a hybrid membrane (PSf) for bisphenol derivatives (BPA) [30]. In the spectra for the BPA-MIP powder and the PSf membrane, infrared bands of 1749 cm^−1^ and 1236 cm^−1^ were assigned to C=O stretching and S=O stretching, respectively; however, for the PSf-HMIP membrane, the authors found that the C=O and S=O stretching bands were shifted towards the higher wavenumbers of 1751 cm^−1^ and 1244 cm^−1^, up from 1749 cm^−1^ and from 1236 cm^−1^, respectively. This observation proved the interaction between the BPA-MIP powder and the membrane scaffold in the PSf-HMIP membrane.

The FTIR spectrum for MIP alone can be seen in our previous study, that of Zakaria *et al.* [31], which showed a broad peak of OH stretching at 3452 cm^−1^, CH stretching at 2960 cm^−1^, and C=O stretching vibrations in the 1726 cm^−1^ range. Peaks related to the MIP were not observed in the infrared spectra of CA-MIP (peaks observed in the CA-MIP are related to functional groups in CA) or PS-MIP, although similar peaks were observed by Takeda *et al.* [30], hybridizing BPA-MIP with a PSf membrane.

#### 2.1.2. Scanning Electron Microscopy (SEM) Images of CA-MIP and PS-MIP

**Figure 3:**
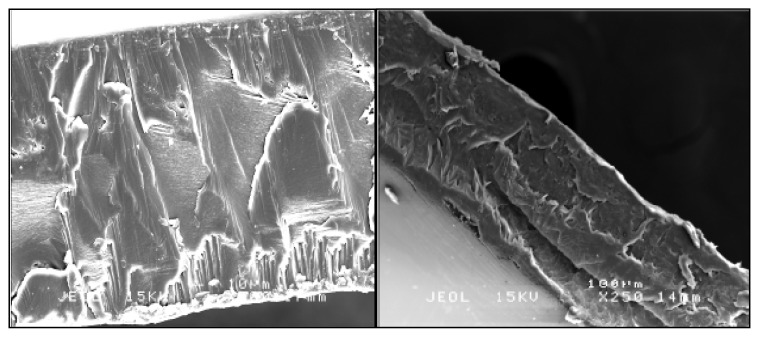
SEM image of the cross section of CA (JEOL 15KV, magnification 500 and cross section 11 mm) and PS (JEOL 15KV, magnification 250 and cross section 14 mm).

**Figure 4:**
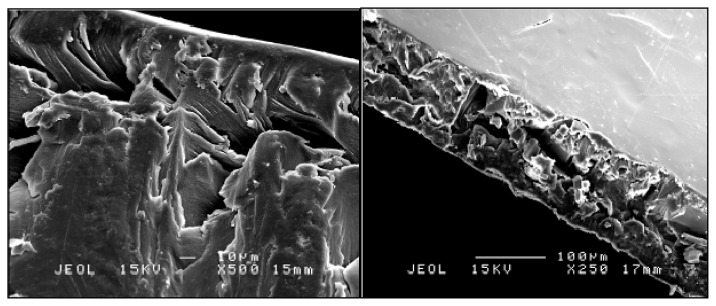
SEM image of the cross section of CA-MIP and PS-MIP.

Imprinted membranes, targeted at 2,4-DNP, were characterized using SEM imaging analysis. Figure 3 shows the morphology of the cross section of CA and PS membranes: The CA and PS membrane cross sections are smooth, compared to that of the MIP membrane (Figure 4), which is more porous. This proves that hybridization of the imprinted polymer particle was successful in each polymer. Takeda *et al.* [30], who hybridized a bisphenol derivative in a PSf membrane, observed the same effect, obtaining a porous structure when hybridizing BPA-MIP in the PSf membrane. The thickness of the CA and CA-MIP membranes was around 150 μm, and the thickness of the PS and PS-MIP membranes was around 200 μm.

### 2.2. Sorption of 2,4-DNP by CA-MIP and PS-MIP

#### 2.2.1. Effect of pH

**Figure 5:**
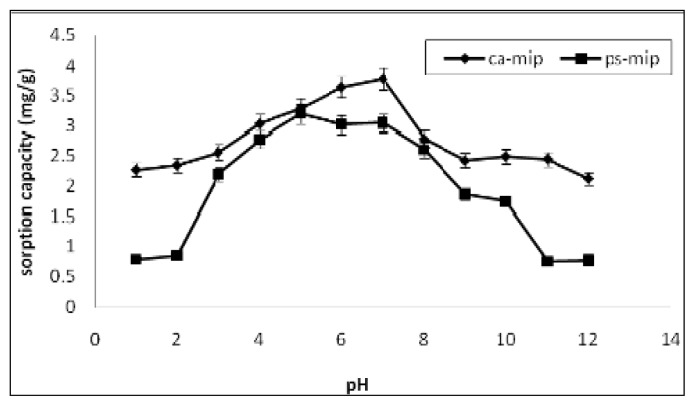
Effect of pH on the removal of 2,4-DNP by CA-MIP and PS-MIP.

Figure 5 shows the effect of pH on the adsorption of 2,4-DNP by CA-MIP and PS-MIP. For CA-MIP, as the pH value of the solution increased, the adsorption efficiency gradually increased and attained a maximum value at pH 7 and then decreased in the basic pH range. This may be because the main binding force between CA-MIP and the 2,4-DNP molecule is an ionic interaction and a hydrogen bond. Only in the approximately neutral solution is the concentration of H^+^ and OH^−^ minimal, which lessens their influence and the binding interaction between the target molecule and sites.

The sorption capacity of the other sorbent, PS-MIP, is lower than that of CA-MIP, except at pH 5. The greater sorption of 2,4-DNP onto CA-MIP, relative to PS-MIP, could be attributed to the stronger interactions between CA-MIP and 2,4-DNP or the nitrophenolate ion. Due to the nitro group, 2,4-DNP is a polar molecule and has a pKa of 7.15. The interaction between CA-MIP and the adsorbate is a cation–dipole interaction, and also involves van der Waals forces. Furthermore, hydrogen bonding is possible between the hydroxyl group of 2,4-DNP and the carbonyl groups in the sorbent.

#### 2.2.2. Kinetics of the Sorption of 2,4-DNP by MIP and a MIP Membrane

**Figure 6:**
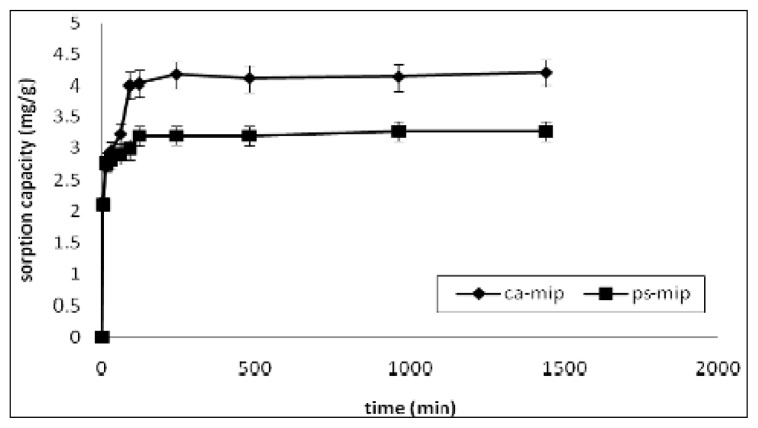
Sorption rates of 2,4-DNP by CA-MIP and PS-MIP. The initial concentration of 2,4-DNP is 10 mg/L.

The kinetics of the sorption process is an important parameter in evaluating sorbent quality and the efficiency of removal. The MIP membrane’s sorption capacity of 2,4-DNP was measured as a function of time (Figure 6). The sorption of 2,4-DNP for CA-MIP and PS-MIP reached equilibrium in one hour and two hours, respectively. The rate of binding was rapid compared to that of other methods, as in using alginate gel beads, where the sorption kinetics for nitrophenol reached equilibrium at 72 h [32]. Another example is the recovery of nitrophenol using the adsorbent Amberlite IRA-900 modified, where equilibrium was achieved after 150 min [2].

**Figure 7:**
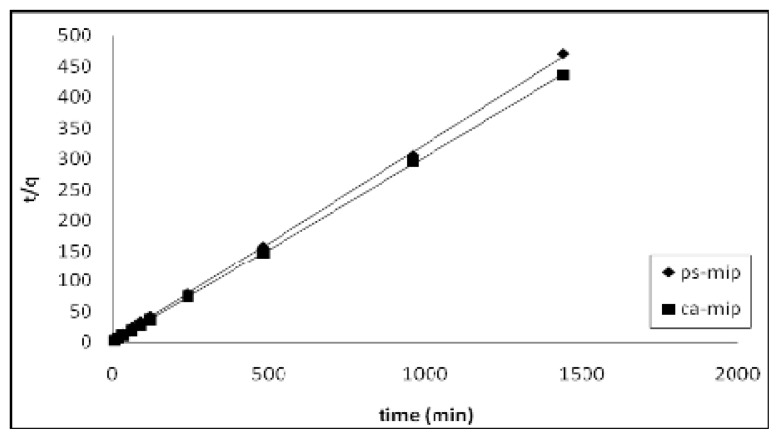
Second-order kinetic plots for the sorption of 2,4-DNP by CA-MIP and PS-MIP.

**Figure 8:**
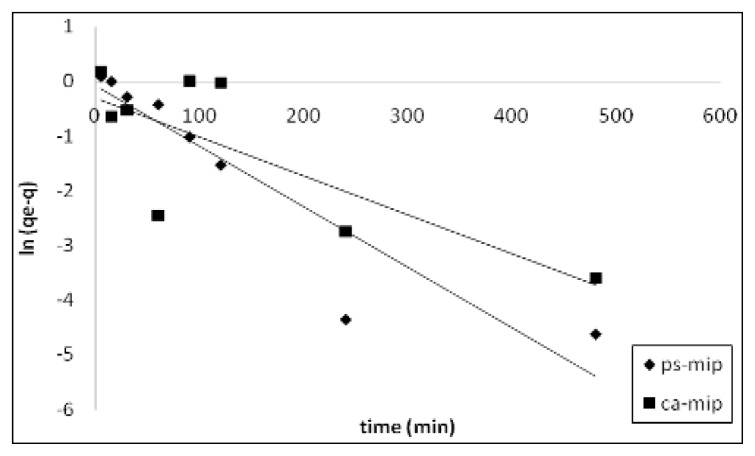
The first-order kinetic plots for the sorption of 2,4-DNP by CA-MIP and PS-MIP.

The removal of 2,4-DNP was mathematically expressed in terms of sorption kinetics. The corresponding parameters of the different kinetic models were determined by linear regression of the plots and are shown in Table 1. The correlation coefficient *r*^2^ of the plots indicates that the pseudo–second-order kinetic model (Figure 7) better describes the sorption of 2,4-DNP by CA-MIP and PS-MIP than the first-order kinetic model (Figure 8).

#### 2.2.3. Selectivity Study

**Figure 9:**
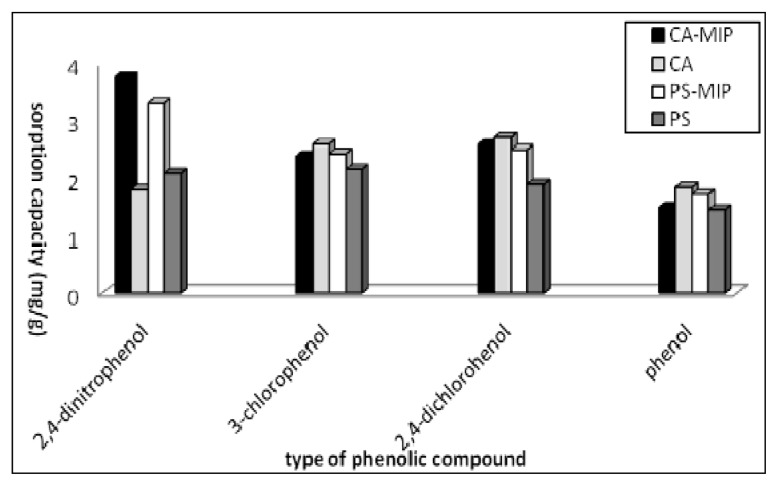
Selectivity study of the removal of 2,4-DNP by CA-MIP and PS-MIP in the presence of various phenolic compounds.

A selectivity study was carried out to remove the 2,4-DNP from an aqueous solution, even in the presence of phenolic compound interference. For this purpose, 2,4-dichlorophenol (2,4-DCP), 3-chlorophenol (3-CP), and phenol, were selected as potential interferences. As shown in Figure 9, the best binding results were obtained for 2,4-DNP. Although 2,4-DCP, 3-CP, and phenol, showed some affinity towards CA-MIP and PS-MIP, it was lower than that of 2,4-DNP. This finding indicates that the MIP membrane had greater molecular recognition towards its template. The reason for this is that the MIP can recognize its template molecule due to the existence of memory cavities of fixed size and shape, binding sites, and specific binding interactions between the target molecule and sites [33]. Zakaria *et al.* [31] also compared the selectivity of synthesized MIP and non-imprinted polymer (NIP), where the synthesized MIP showed greater binding capacity towards 2,4-DNP than the NIP did (Table 2).

## 3. Experimental Section

### 3.1. Chemicals

Acrylamide and 2,4-DNP were supplied by Fisher Chemical, and Sigma-Aldrich, respectively. Benzoyl peroxide, acetic acid, and tetrahydrofuran were supplied by R & M Chemical Technologies. Acetonitrile and methanol were supplied by HmbG Chemical. Ethylene glycol dimethacrylate (EDGMA) was supplied by Fluka Chemical. The CA and polystyrene (PS) were supplied by Acros Organic. Distilled water was used throughout the experiment.

### 3.2. Instruments

The MIP membrane was characterized by FTIR with a Perkin Elmer 1600 Spectrophotometer and SEM JEOL JSM 6700 and JEOL JSM 6400. The sorption experiments were carried out using Varian Cary WinUV spectrophotometer.

### 3.3. MIP Synthesis

The MIP was prepared using a non-covalent approach. The template, 2,4-DNP (DNP, 1.0 mmol), was dissolved in a beaker of acetonitrile (30 mL). The functional monomer acrylamide (5.0 mmol), the cross-linker EDGMA (15 mmol), and the initiator benzoyl peroxide (1.6 mmol) were then added to the flask. After 10 min of degassing and nitrogen purging, the flask was sealed and the contents allowed to polymerize in a water bath at 70 °C for 24 h. The bulk polymers obtained were crushed, ground, and sieved into regularly sized particles between 80 μm and 100 μm. Finally, the MIP particles were extensively washed with distilled water to remove any unreacted monomer or diluents. The polymer was then washed with 1:2 (*v*/*v*) methanol–acetic acid until the template was completely removed.

### 3.4. Preparation of the MIP Membrane

The MIP particles were hybridized in porous membranes of CA and P by a phase inversion process. Tetrahydrofuran was used as a solvent. A total of 500 mg of each polymer, CA and PS, were dissolved in 30 mL of tetrahydrofuran. The MIP particles (1000 mg) were mixed into each polymer solution by stirring within 4 h at 50 °C. The resultant viscous solution was spread on a glass plate and dried overnight at room temperature.

### 3.5. Sorption of 2,4-DNP by the MIP Membrane

Sorption experiments for 2,4-DNP by the MIP membrane underwent characterization by pH, sorption isotherms, and sorption kinetics and a selectivity study. A 2 × 2 cm^2^ piece of MIP membrane was cut and applied to the sorption of 10 mg/L of 2,4-DNP and stirred for 24 h. The concentration of 2,4-DNP in the aqueous solution after the desired treatment periods was analysed using an ultraviolet–visible spectrometer.

#### 3.5.1. Effect of pH

The MIP membrane was stirred for 24 h with 20 mL of 10 mg/L 2,4-DNP at pH values of 1–12. The pH was adjusted with hydrochloric acid or sodium hydroxide. The MIP was then filtered and the final pH was measured.

#### 3.5.2. Sorption Kinetics

For sorption kinetics, 20 mL of 10 mg/L 2,4-DNP solution, at the optimum pH obtained from the previous experiment were stirred with the MIP membrane for various time periods (5 min, 10 min, 20 min, 30 min, 60 min, 120 min, 240 min, 480 min, 960 min, and 1440 min). The MIP membrane was then filtered and the filtrate analysed for 2,4-DNP concentrations with the ultraviolet–visible spectrometer.

#### 3.5.3. Selectivity Study

The selectivity of the fabricated MIP towards phenol, 3-CP, and 2,4-DCP with respect to 2,4-nitrophenol was studied. A solution (25 mL) containing 10 mg/L (of each compound) was mixed together and treated with MIP membrane at room temperature. The concentrations of the phenolic compounds, after treatment, were measured by the ultraviolet–visible spectrometer. The binding capacity was then calculated.

## 4. Conclusions

A MIP membrane was successfully prepared by the hybridization of MIP particles with CA and PS. The MIP was characterized by FTIR and SEM. The maximum sorption of 2,4-DNP by the fabricated CA-MIP and PS-MIP was found at pH 7.0 and pH 5.0, respectively. Kinetics found that the rate of sorption of 2,4-DNP increased rapidly in the initial stage, and then slowed until it reached equilibrium. A pseudo–second-order kinetic model is more suitable to describe the sorption process onto CA-MIP and PS-MIP, based on the correlation coefficient values. The selectivity experiments showed that the MIP is selective towards 2,4-DNP in the presence of 2,4-DCP, 3-CP, and phenol interference. It was confirmed that the shape and size of the template, as well as the strength of interaction between the target molecule and binding sites, determine MIP selectivity.

## Figures and Tables

**Table 1:** Kinetic parameters for the sorption of 2,4-DNP by CA-MIP and PS-MIP.

Kinetic model parameter		Sorbent	

		CA-MIP	PS-MIP
First-order	*k*_1_(min^−1^)	0.233 ± 0.01	0.145 ± 0.01
	*q*_e_ (mg g^−1^)	0.63 ± 0.1	0.84 ± 0.1
	*r*^2^	0.602	0.864
Pseudo–second-order	*k*_2_ (g mg^−1^ min^−1^)	0.370 ± 0.01	0.115 ± 0.01
	*q*_e_ (mg g^−1^)	3.30 ± 0.1	3.10 ± 0.1
	*r*^2^	0.999	0.999

**Table 2:** Parameters involved in the selectivity study of the MIP and NIP towards different kind of phenolic compound [31].

	*K*_d(MIP)_ (mg/g)	*K*_d(NIP)_ (mg/g)	*k*_(MIP)_	*k*_(NIP)_	*k′*
2,4-DNP	3.768	1.71			
Phenol	1.493	1.946	2.534	0.878	2.88
3-CP	1.548	1.728	2.431	0.989	2.46
2,4-DCP	2.169	1.917	1.737	0.892	1.95

Here *K*_d_ is the distribution coefficient and *k* is the selectivity coefficient: $k^{\prime }=\frac{k_{MIP}}{k_{NIP}}$.
